# Semantic memory in developmental amnesia

**DOI:** 10.1016/j.neulet.2018.04.040

**Published:** 2018-07-27

**Authors:** Rachael L. Elward, Faraneh Vargha-Khadem

**Affiliations:** Cognitive Neuroscience & Neuropsychiatry Section, UCL Great Ormond Street Institute of Child Health, 30 Guilford Street, London, WC1N 1EH, United Kingdom

**Keywords:** Semantic memory, Developmental amnesia, Hippocampal atrophy, Neonatal hypoxia-ischaemia

## Abstract

Patients with developmental amnesia resulting from bilateral hippocampal atrophy associated with neonatal hypoxia-ischaemia typically show relatively preserved semantic memory and factual knowledge about the natural world despite severe impairments in episodic memory. Understanding the neural and mnemonic processes that enable this context-free semantic knowledge to be acquired throughout development without the support of the contextualised episodic memory system is a serious challenge. This review describes the clinical presentation of patients with developmental amnesia, contrasts its features with those reported for adult-onset hippocampal amnesia, and analyses the effects of variables that influence the learning of new semantic information.

## Highlights

• Profile of patients with developmental amnesia and dissociations in cognitive memory.
• Variables affecting semantic learning in developmental amnesia.
• Differences between adult-onset versus early-onset amnesia.

## 1 Introduction

A conundrum exists in the neurodevelopmental literature where patients with early hippocampal damage exhibit profound amnesia for their life events, but are able to form semantic memories which generalise across those same events. This is illustrated with an anecdote from patient Jon, a well-documented case of developmental amnesia (DA - [1], [2]. Jon sustained severe bilateral hippocampal damage as a result of hypoxic-ischaemic events that occurred when he was a neonate. Throughout his childhood and adult life, he has had difficulty remembering episodes from his past. Jon frequently visits our laboratory in London. To do so, he travels to an underground train station nearby, then takes the lift to the street level and walks the remainder of the journey. On one such visit, the lift at the underground station was out of order, and Jon had to climb the 171 steps to the surface (the equivalent of some 14 floors). When he arrived at the laboratory, he had no recollection of having climbed the stairs, and confidently reported that he had taken the lift as normal. Jon was questioned about his memory of this event; “How do you know that you took the lift today?”. Jon declared: “I always take the lift!”. Why is Jon so confident that he *always* takes the lift, when he has no episodic memory of doing so? If Jon has no recollection of his life events as they occur, how does he learn what he typically does?

Theoretical models of human memory posit that the semantic memory system and the episodic memory system are dissociable (see Squire and Zola [3] for an alternative view), such that memory for non-contextual facts (e.g. “I always take the lift”) are supported by a different set of brain regions than memory for episodes that are bound in a specific spatial-temporal context (e.g. “I climbed the stairs today because the lift was out of order” [4], [5], [6]. As a consequence of his hippocampal damage, Jon has a severe deficit in his episodic memory abilities, but given the apparent integrity of his parahippocampal cortex, the putative neural substrate for processing non-contextual information, his semantic memory system is relatively intact. Consequently, Jon, and other patients with this developmental form of amnesia, are able to acquire a remarkable repository of semantic information about the world such that they are up to date with current affairs, major news items, and new discoveries, etc. This is all the more impressive considering that their dictionary of factual world knowledge is gradually amassed in the presence of early hippocampal damage, long before any overt signs of memory ability has emerged. Of note, Jon and other patients with DA, appear to use their good semantic memory to produce a reasonable response to questions relating to specific episodes, thus giving the impression that they do not have a memory problem at all [7], [8]. In this way, patients with DA appear to use their knowledge of the world to construct a general representation of events in the absence of the ability to reconstruct the specific experiential aspects of the episodes. The dissociation between episodic and semantic memory described above is now reported in both large group studies of patients with DA [9], [10] and several single case studies [11], [12], [13], [14], [15], [16], [17].

The question still remains however: “*how* can patients with DA acquire semantic information in the presence of hippocampal damage?” Theoretical accounts of semantic memory formation begin with the memory of a unique experience (the memory of which is dependent on the hippocampus) and these experienced memories subsequently become consolidated and stored in the cortex over time [18], [19], [20] see also [21], [22]. As a result of this transformation, the “gist” of the episode is retained, and the factual aspects are extrapolated into the semantic system, with the unique features of the individual events gradually lost over time. The gist of memoranda stored in the cortex are available to consciousness and can be retrieved and “declared” at will. This progression from hippocampal-dependent memory encoding to cortically-stored memory retrieval implicates a crucial role for the hippocampus in the formation of semantic memories. If this is so, then young people with early hippocampal damage should struggle to learn language, and should be as impaired on semantic memory tests as they are on episodic memory tests, yet this is not the profile of DA.

A cognitive model that is compatible with ontogenetic development of memory in humans is proposed by Tulving [6], [23]. This posits that cognitive memory is organised hierarchically into four systems (*viz,* perceptual learning, semantic learning, working and/or short term memory, and episodic memory; see Table 1 below). Each system contains a set of subsystems and a retrieval mode. An important feature of this model relevant to the issue of semantic memory development in DA is that the operation of each system does not depend on the higher systems operating. Conversely, the operation of the later-emerging systems is dependent on, and supported by, the operation of the earlier-emerging ones.

**Table 1:** Adapted from Tulving [23]. Major categories of human learning and memory.

System	Other Terms	Subsystems	Retrieval
Procedural	Nondeclarative	Motor skills	Implicit
		Cognitive skills	
		Simple conditioning	
		Simple associative learning	
PRS	Priming	Structural description	Implicit
		Visual word Form	
		Auditory word Form	
Semantic	Generic	Spatial	Implicit
	Factual	Relational	
	Knowledge		
Primary	Working	Visual	Explicit
	Short Term	Auditory	
Episodic	Personal		Explicit
	Autobiographical		
	Event Memory		

Tulving [23], describes the process-specific relations among these categories through the SPI model (*viz,* Serial, Parallel, Independent Model). According to this, information is encoded into the cognitive memory system in a serial order (*viz,* in time), stored in multiple systems and subsystems in parallel (e.g. during perceptual learning, multiple copies of the same original percept are stored in different sensory modalities), and retrieved independently from different repositories depending on the needs of the situation (e.g. the picture of a plate of spaghetti might evoke the gustatory stored copy of the meal).

**Fig. 1:**
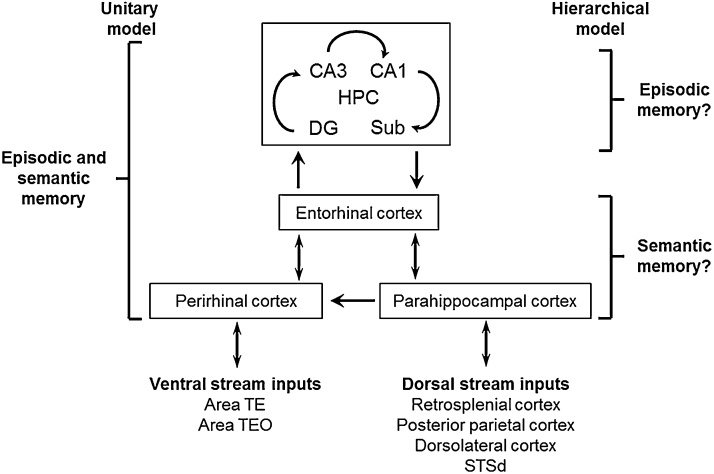
Schematic diagram of the connections of the medial temporal hippocampal network modified from Mishkin et al. 1997 [5]. The areas listed under ventral and dorsal streams are those providing the strongest inputs to the perirhinal and parahippocampal cortices, respectively.

The ontogenetic unfolding of cognitive abilities from the SPI model readily maps onto the neuroscience-based model of hierarchical organisation of cognitive memory proposed by Mishkin et al. [5]. Here, the hippocampus is the hub and the recipient of reciprocal projections from the sensory processing streams [24]. Sitting at the apex of the hierarchy, the hippocampus binds the temporal and spatial features of memoranda in preparation for storage. A hierarchically-organised medial temporal lobe system with the hippocampus at the apex allows for *single* dissociations in cognitive memory such that a lesion affecting the hippocampus compromises contextualised episodic memory selectively, but spares the independent encoding, storage and retrieval of decontextualized factual memory, as in patients with DA. Fig. 1 below illustrates the neural substrates of the hierarchical model along with that of the unitary model, for the dorsal and ventral processing streams of the visual system. In theory the model is applicable to all sensory modalities.

## 2 Development of memory in the presence of hippocampal damage

The majority of patients with DA sustained their bilateral hippocampal lesions during the neonatal period consequent to hypoxic-ischaemic episodes. Despite the severity of these episodes, they did not sustain any neurological damage [25]. Once they recovered from the catastrophic circumstances of their hypoxic events, they developed normally achieving their milestones for walking, speech and language, vocabulary knowledge, and early educational skills to age-appropriate standards. They recognised familiar people and objects, and were able to learn their daily routines. As they grew older, however, these children came to medical attention because they were unable to remember instructions, routinely lost their belongings, failed to deliver messages, got lost in new surroundings, forgot important events (e.g. birthday parties, family holidays, special trips, etc.), and failed to remember the subjects covered in class.

Viewed within a developmental framework, the strengths and weaknesses that emerge with increasing age in patients with DA strongly suggest that the *normal* trajectory for the acquisition of a wide range of cognitive functions, such as vocabulary knowledge, conceptual language, grammar, reading, writing, number skills, is not dependent on the integrity of the hippocampus. Furthermore, to the extent that these cognitive abilities, and presumably the neuronal networks that support them, continue to progress throughout childhood and adolescence, it appears that their maintenance is not hippocampus-mediated either.

**Fig. 2:**
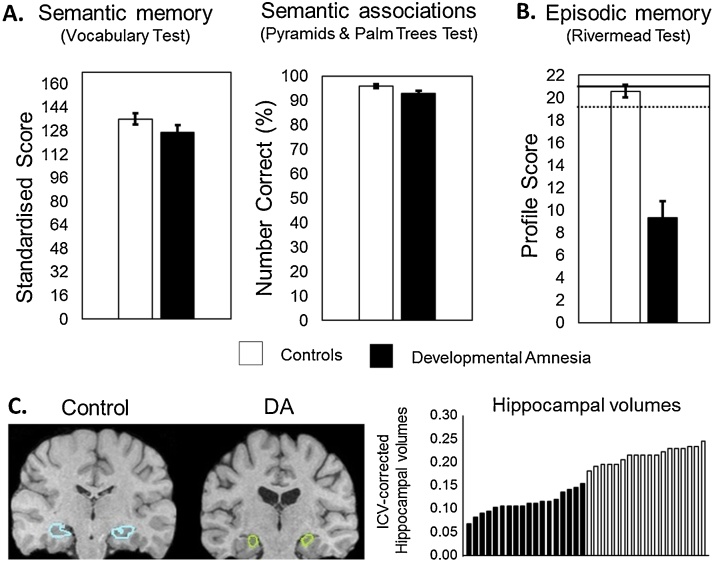
A. Semantic memory scores for DA patients and controls. B. Episodic memory scores for DA and controls. C. Hippocampal volumes for patients with DA and controls.

In a study reporting on the largest series to date of patients with DA (N = 18; [9], neuropsychological assessments revealed a striking dissociation between intelligence and memory. Whereas the mean intelligence quotient of the group yielded the standardised score of 95 (normal range compared to both matched controls and the standard population mean; x = 100; sd, 15), the mean memory quotient was 61 (exceptionally low range) compared to the matched control groups’ standardised mean of 108. Similarly, the standardised scores for working memory, literacy and numeracy were all in the normal range and not significantly different from the means of matched controls. More impressive, however, were the scores achieved on two measures of semantic memory, the first assessing vocabulary knowledge tested through word-picture matching (British Picture Vocabulary Test), and the second evaluating word-picture semantic associations (Pyramids and Palm Trees Test). On both of these tests, the DA group achieved very high scores (see Fig. 2A). In contrast to such high-level performance on tests of semantic knowledge, the DA group was severely impaired on a test of episodic memory (Rivermead Behavioural Memory Test) which evaluates memory for everyday events, such as remembering a name, the location of a belonging, a story, a simple route around the room, and delivering a message, etc. (Fig. 2B). Fig. 2C **-** Left, shows a snapshot of the integrity of cortical areas outside of the atrophied hippocampi in a patient with DA, while Fig. 2C - Right illustrates the severity of the hippocampal volume reduction in the DA group relative to controls (range = 28–62% below normal), a reduction that parallels the severity of the episodic memory impairment.

The neuropsychological and the neuroimaging profiles, along with the medical history of early hypoxic-ischaemic injury are characteristic features not only of this large group of patients with DA, but also of single case studies reported to date (e.g. [2], [12], [15], [16], [17]. Overall, these findings indicate that semantic memory may develop normally throughout childhood and adolescence in the presence of early, and relatively selective damage to the hippocampus.

## 3 Comparison with adult-acquired hippocampal damage resulting in amnesia

A seemingly comparable dissociation, at least in pattern if not in degree, between semantic memory and episodic memory has been demonstrated in adult-acquired hippocampal damage resulting in amnesia. One such case, Patient PS, was described by Verfaellie et al. [26]. Like Jon, patient PS has a selective lesion of the hippocampus caused by anoxia, but PS’s anoxic event occurred at the age of 40, long after her episodic and semantic memory systems had developed (whereas Jon’s anoxic event had occurred in the perinatal period before either memory system had developed). Administered a series of neuropsychological tests, Patient PS showed floor level performance on tests assessing episodic memory, but relatively normal performance on tests of semantic knowledge and vocabulary. At first glance, it appeared that the same semantic/episodic dissociation documented in patients with DA could also emerge following adult-onset hippocampal amnesia. However, in this case, semantic memory was first assessed through retrieval of stored knowledge that dated back to decades before the hippocampal damage had occurred. This suggests that the hippocampal injury did not interfere with stored semantic representations that had accumulated over the years. However, when new semantic information that had not previously existed was assessed (e.g. new words that had entered the lexicon in the years since the hippocampal injury, or knowledge of famous people who had gained popularity since the anoxic event), PS’s performance was impaired relative to controls [27]; see also [26]. These findings indicate that in contrast to reports in patients with DA, acquisition and/or retrieval of *new* post-injury semantic memory is compromised after selective adult-onset hippocampal damage.

Notwithstanding the caveat that measures of episodic memory and semantic memory are not equated, and therefore not quantitatively comparable, a possible explanation for the difference is that patients with adult-onset hippocampal amnesia continue to rely, unsuccessfully and often frustratingly, on their damaged hippocampal-cortical network for mnemonic processing and retrieval. In contrast, patients with DA, who as neonates or young children have maximum plasticity and reorganizational capacity, develop their memory system in a way that optimises their semantic learning even in the presence of severe hippocampal damage [28]. Given that in patients with DA all vocabulary and world knowledge is acquired in the years *after* the hippocampal injury, and that these patients have at least age-appropriate (if not supra-age level) banks of knowledge, it must be concluded that the hippocampus does not play a crucial role in the building up of semantic memory when the injury occurs very early in life. This implies that the age at which the hippocampal injury occurs may be an important determinant of the pattern and extent of rescued capacity for semantic memory development. As a result, the semantic/episodic distinction may be more complex in adult-onset hippocampal amnesia than in DA. This literature can be difficult to interpret, however, as few patients suffer selective damage to the hippocampus in adulthood without also sustaining damage to the surrounding medial temporal structures. Furthermore, adult amnesic patients have not been compared directly to DA patients using the same paradigms (and controlling for the extent of pathology). Based on the available data, however, indications are that patients who acquire hippocampal damage in the neonatal period or in childhood are better able to rescue semantic memory, but not episodic memory, compared to those who acquire the amnesia-inducing hippocampal injury during adulthood.

## 4 Autobiographical facts

We began this review with an anecdote about patient Jon which showed that he is able to describe facts about his life in a way that resembles semantic memory for his personal experiences, but is not able to describe the events that underlie these facts. To better understand this phenomenon, researchers have investigated such “autobiographical facts” in patients with amnesia [14], [29], [30], [31]. Maguire et al. [29] conducted an fMRI study with Jon to examine different types of episodic and factual memory (*viz,* autobiographical and personally experienced, autobiographical facts, public events, general knowledge) that might be supported by different patterns of brain activations. Through questionnaire and interviews with Jon and his family, information about events in Jon’s life was obtained. For a very limited number of autobiographical events, Jon was able to report a genuine episodic memory, but for many others Jon “knew” (as a fact) that the event had occurred, but he could not “recollect” the episode as a personally-experienced event.

Inside the MRI scanner, Jon was reminded of these autobiographical events and activity was contrasted between the two types of memory experience that were ascertained in the interview (episodic memory vs. fact memory). Jon showed greater activation in the hippocampus for the autobiographical events that he remembered than for those that he merely knew. It is difficult to make the same comparison in controls because details about events that have been personally experienced are readily recollected, rather than “known” to have occurred. Despite the caveats that Jon activates a bilateral rather than a left-lateralised cortical and medial network, and his autobiographical memoranda are comprised of some episodes that are remembered and some that are known, the data nevertheless suggest that Jon is able to retrieve semantic memories for his life events which do not depend on the same level of support from the hippocampus as recollected episodes.

In a similar case, Picard et al. [14] reported an 18-year old girl named Valentine who developed amnesia in childhood following a period of anoxia at birth. A neurological exam at 13 years of age revealed that Valentine had normal intelligence, language, semantic and working memory, but had great difficulty with episodic memory, presumably because of her bilateral hippocampal atrophy. Valentine was asked to produce various semantic autobiographical memories (e.g. the names of teachers) and episodic autobiographical memories (e.g. personally experienced events). Consistent with the pattern of data evidenced in patient Jon, Valentine recalled fewer episodic autobiographical memories than a group of age-matched controls, but her semantic autobiographical memory was unimpaired. Remarkably, Valentine’s semantic autobiographical memory score was significantly better than controls for the most recent memories. Clearly, Valentine’s ability to learn semantic facts about her life was not noticeably impaired. Furthermore, it reportedly did not take many years for Valentine to consolidate these semantic memories into the ‘putative’ cortical memory system − rather, these memories seem to have formed readily despite her hippocampal amnesia. This case refutes the idea that semantic memories depend on support from the hippocampus for years (or decades) prior to consolidation into the cortex.

Retrieval of autobiographical facts may be compromised, however, when these facts are associated with a specific spatio-temporal context. Grilli and Verfaellie [31], distinguished between autobiographical facts that are classified as “experience near” (e.g. I was in New York in September) versus those that are “experience far” (e.g. I have a son). Grilli and Verfaellie [31] asked adult patients with MTL lesions to make “I am” statements and then asked them to elaborate on each of those statements. Patients with MTL lesions produced fewer experience-near autobiographical facts than controls, but not fewer experience-far autobiographical facts. These results raise the possibility that memory for facts about one’s life experiences may not be substantially different from memory for semantic information about the wider world. To the extent that personally-relevant facts are tied to specific episodic memories, then retrieval of those memoranda depend on the hippocampal system, but personal facts that are not associated with a unique experience may be formed and retrieved independently of the episodic system, and, by implication, independent of support from the hippocampus. This interpretation is supported by a *meta*-analysis by Martinelli et al. [32] which showed that episodic autobiographical information was associated with activation in the hippocampus but that semantic autobiographical information was not. Semantic autobiographical information was instead associated with a network of cortical areas including the parahippocampal cortex.

## 5 Semantic learning in developmental amnesia

Having ascertained that patients with DA have an impressive capacity for acquiring semantic knowledge, it remains unclear how this information is acquired. To our knowledge, only a handful of prospective studies have investigated the acquisition of new semantic information under laboratory conditions where variables affecting encoding, storage, and retrieval processes could be systematically manipulated [2], [8], [13], [33] along with some unpublished research currently in progress in our laboratory spanning a period of a decade and a half.

An important caveat for this research is that control participants have access to two systems when acquiring new information: The semantic system and the episodic system. Healthy controls can re-experience a prior learning episode in which semantic information was obtained (e.g. they can remember a time that they dissected a frog in school, or remember watching an interesting documentary). In contrast, patients with hippocampal damage cannot remember their learning episodes, but they do have a semantic system which contains de-contextual knowledge about the world into which novel semantic information may be assimilated. As a consequence, it cannot be expected that patients with DA can recall newly- acquired information as readily as healthy controls, who more than likely use the additional support of episodic memory to recall recently acquired facts.

### 5.1 Semantic learning through viewing of film clips

**Fig. 3:**
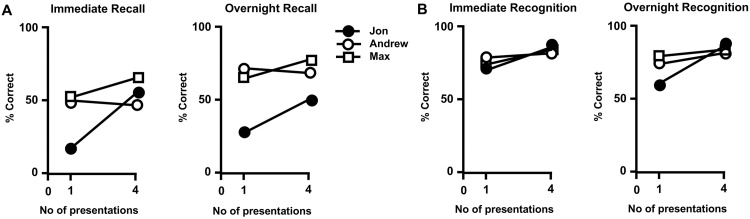
Recall (A) and Recognition (B) scores from The Newsreel Task modified from Baddeley, Vargha-Khadem and Mishkin [2].

During the course of interviews with Jon and his family, it emerged that much of the acquired world knowledge of Jon was based on regular exposure to television news programmes and newspapers. The first study, conducted by Baddeley and colleages [2], used this medium to simulate the way Jon might acquire information about world events from news reports. Jon (and his two age- and IQ-matched controls) were presented with Newsreel footage of events, the content of which predated by several decades the birth of the participants (e.g., the Hindenburg disaster; the coronation of King George VI). In this study, the variables of delay (immediate versus 24 h delay after overnight sleep), number of presentations (1 versus 4 repetitions), and retrieval process (recognition versus recall) were manipulated. Fig. 3A illustrates the performance of Jon and his controls using cued recall to retrieve the encoded and stored information. This shows that after one exposure, Jon’s immediate recall of the relevant information was much less compared to that of his controls. However, for the footage that was presented 4 times, Jon’s immediate recall substantially improved and rose to the same standard of his controls. After 24 h, Jon’s delayed recall of the footage presented only once was greatly reduced, almost to the level seen after immediate recall, but this substantially improved when the footage was repeated 4 times, although the improvement did not match that of his controls. These results suggest that whilst Jon learned the content of the videos at a slower rate than his controls, he benefitted from repetition for retrieval of semantic information both in immediate and delayed conditions, at least over the 24-h delay period.

When comparable news footage was assessed through recognition however (participants had to select the correct semantic details from a list of alternatives), Jon’s performance closely resembled that of controls (Fig. 3B). Here, the data show that after both 1 and 4 exposures, Jon encoded and retrieved the semantic contents of the two videos to the same standard as his controls in the immediate condition. After overnight sleep, Jon’s performance reduced only slightly for the footage shown once, but not for that presented 4 times.

At least two important conclusions emerge from this data set. First, Jon’s retrieval through recognition is at a high level and comparable to that of controls. It is therefore possible that some aspects of new semantic learning in DA maybe attributable to the integrity of the recognition process, which may aid the acquisition and consolidation of decontextualized world knowledge through the implicit sense of familiarity, even in circumstances where the information is not available to recall. The dissociation between recognition and recall has been confirmed as an important feature of DA, and shown to prevail in a group study directly comparing the two processes in 12 patients with DA and their matched controls [40]. Furthermore, recall, but not recognition, has been shown to be correlated with the degree of hippocampal atrophy [34].

The second conclusion is that repeated exposure to new semantic information improves recall suggesting that while repetition may be important for consolidation, episodic recollection of that information may not be critical for consolidation to occur. Crucially, the data suggest that the hippocampus is important for fast acquisition of new information that is subsequently available to recall; with repeated exposure, however, a semantic representation may emerge even in the absence of hippocampal support.

### 5.2 Learning new information through repeated recall of text

The second prospective study was initiated because of concerns that despite their high levels of intellectual ability, patients with DA struggle to learn new factual information within the classroom setting. On the assumption that a sufficient number of repetitions under controlled conditions might enhance recall, the study involved 5 phases of learning separated by one or more years between each phase. Only the first learning phase of the study will be described [35].

Here, the aims were to (a) track learning from trial-to-trial after 6 repetitions and test with cued recall, (2) determine the effects of short delay (30 min) versus long delay (one week) on recall; and (3) document the status of learned material after long delay based on cued recall versus cued recognition. Four written texts with 35 chunks of information of equal length, complexity, and number of items, matching on word length and reading level were prepared. Six learning trials were presented wherein written paragraphs were accompanied by audio recordings of the text. After a 3 min delay during which working memory was blocked by engaging in conversation, cued recall requiring written answers to questions were requested. Cued recall was also obtained after a short delay of 30 min, and then after a long delay of one week. Three male patients with DA, including Jon, were assessed along with six age-, gender-, and IQ-matched controls. Three controls completed only two learning trials, while three others learned the text to the criterion of 80% accuracy.

**Fig. 4:**
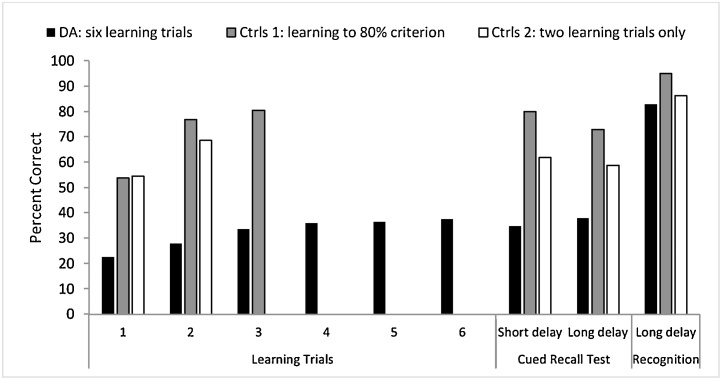
Rate of Learning of new semantic information from text and subsequent cued recall and recognition memory performance in patients with DA and two control groups [35].

Results shown in Fig. 4, revealed that patients with DA learned approximately 35% of the relevant information by the third learning trial; thereafter, their performance plateaued up to the sixth learning trial. Interestingly, the patients’ cued-recall remained at the same level as the last learning trial after both short and long delay, with no indication of forgetting over time. Each of the control groups scored about 10% lower at delayed recall relative to their highest level of learning, with minimal forgetting indicated after the short delay. The recognition accuracy of the patients with DA was above 80% and not significantly different from that of controls. Once again, these data confirm that whilst the rate of learning of semantic information in patients with DA is much lower than normal, they do encode and simultaneously consolidate a small percentage of the relevant information and retrieve it through cued-recall after a long delay. Importantly though, the stored information is accessible through recognition with high fidelity and shows no sign of forgetting. These results are on the whole consistent with those reported by Guillery-Girard and colleages [13] on two children with developmental amnesia. The challenge remains, however, as to how can such stored information that is accessible through recognition find expression via self-initiated recall.

### 5.3 Learning new information through repeated recognition trials

**Fig. 5:**
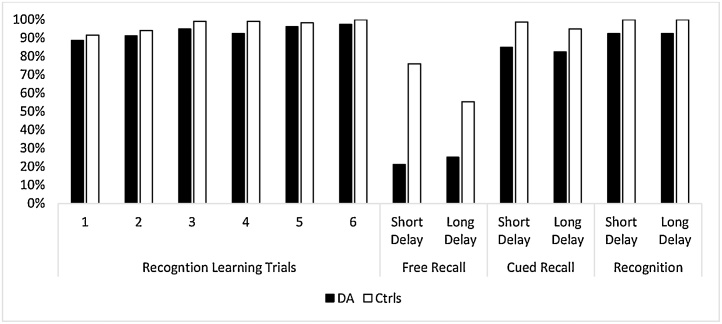
Rate of Learning of new semantic information from videos and subsequent cued recall and recognition memory performance in patients with DA and controls, [37].

The first step towards addressing this challenge is through utilisation of the impressive recognition ability of patients with DA. We reasoned that new learning in developmental amnesia may be improved via repeated recognition tests, rather than cued-recall tests. Using the same texts described for the experiment by Limond et al. [35], see 5.2, & Fig. 4), we designed four videos depicting pictorial content to accompany the texts. These videos were presented over six learning trials to four patients with DA and a group of six controls [37]. Each viewing of the video was immediately followed by a recognition test containing twenty questions about the contents and 4-alternative choices for each question (e.g. Which group of people thought that mistletoe was sacred? (i) The Ancient Druids; (ii) Medieval Priests; (iii) Early Mariners; (iv) Tudor Farmers). The data showed that patients performed close to the level of healthy controls on the multiple-choice recognition tests of the learning trials. Of note, neither group reached 100% accuracy (Fig. 5).

Memory for contents of two videos was subsequently tested after an overnight delay (short delay) while that for the other two was tested after a delay of multiple days up to one week (long delay). Three types of memory tests were subsequently conducted: free recall, wherein participants were asked to report any information from the videos; cued recall, wherein participants were asked the same 20 questions from the learning trials but without any multiple-choice answers; and a recognition test identical to that used during the learning trials (i.e. 4-alternative multiple-choice answers). As in the experiment by Limond et al. [35], patients with DA struggled to free recall the information from the videos, and became upset when they were unable to remember the contents. However, they showed remarkably good performance during cued-recall (see Fig. 5). Using this recognition-based learning paradigm, patients recalled 85% of the semantic information that had been presented in the videos, compared to just 35% in the previous version of the experiment that used recall-learning trials. These data suggest that repeated tests using multiple-choice recognition can support the long term formation of new semantic memory despite the newly-acquired memoranda being unavailable to free recall.

Another interesting finding in this study is that free recall scores of healthy controls fell from 75% after short delay to 50% after long delay. In contrast, the patient group showed no evidence of forgetting over the same delay periods. This suggests that although the DA patients are able to recall only a small amount of the newly acquired information, they nevertheless retain the semantic memories that are formed well enough to access through free recall, with these being relatively resistant to forgetting over time.

**Fig. 6:**
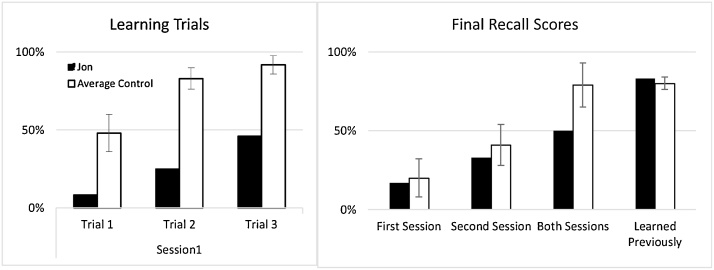
A. Rate of learning of facts in Patient Jon and controls (data from B. Final recall scores for facts learned in the previous learning sessions and knowledge of facts learned prior to the study [8].

In another prospective, semantic learning study in developmental amnesia, Gardiner et al. [8] investigated the acquisition of semantic memory in Jon and a group of controls over a twelve-week period. At the outset of the study, Jon’s standard of general knowledge was compared with that of 8 undergraduate university students and Jon performed slightly better than the university-educated control group. This emphasises the point that Jon, much like other patients with DA, has acquired world knowledge to standards similar to those of well-educated control participants despite struggling with formal education. Gardiner et al. [8], then taught the participants additional facts that they did not know prior to the start of the experiment. This new learning was conducted over two sessions spanning a period of 12 weeks. As in the previous study [35], Jon’s rate of learning differed markedly from controls (see Fig. 6A). Whereas control participants required only three learning trials to reliably recall the relevant information, Jon recalled only half of the new facts that had been presented to him over six learning trials (the max number of presentations permitted by the design). In addition, Jon displayed “intertrial forgetting” wherein he would respond correctly on one learning trial, but respond incorrectly on the subsequent learning trial. Therefore, over the course of a single learning session, Jon did not rapidly form semantic memories in the same way as healthy controls. Once again, these data support the notion that the hippocampus is important for the recall of new information that is acquired rapidly, and when this system is compromised the acquisition of new semantic facts becomes slower, and less reliable.

Twelve weeks after the first learning session, all of the participants returned for a final recall test. Jon’s fact recall was remarkably similar to that of controls. He could recall 17% of items that had been presented in the first learning session (compared to the controls’ recall of 20%, sd = 12) and 33% of facts learned in the second learning session (compared to the controls’ recall of 41%, sd = 13; Fig. 5B). Using a Crawford’s one-sample *t*-test, neither recall scores of Jon are significantly different from those of the controls (max t = 0.29). Therefore, although Jon’s rate of learning was markedly slower than that of controls over the learning trials, after a delay period spanning several weeks, Jon’s ultimate memory for the newly-learnt facts was close to the same level as controls. It is important to note, however, that Jon’s recall scores did not improve over the delay period such that he caught up to the level of controls. Rather, the recall scores of the controls declined rapidly over the delay period and fell to levels similar to those of Jon. Together, the data presented in Fig. 4, Fig. 5, Fig. 6, suggest that despite a low rate of acquiring novel facts, patients with DA show little sign of forgetting newly-learnt information over time, in contrast to controls who appear to lose some of the novel memoranda.

The question then arises as to why healthy controls forget so much of the newly-acquired semantic information over the delay period. This may be due to the caveat described at the beginning of this section, that is, controls have access to episodic memory to support the recall of recently acquired semantic facts. However, hippocampal memories are thought to be vulnerable to forgetting over time, whereas semantic memories by contrast are relatively robust to forgetting over time and are more vulnerable to interference [36]. During the learning trials of the Gardiner et al. [8] study, therefore, controls may have had inflated semantic memory scores because they were able to use episodic memory to support their learning of novel semantic information. With the passage of time, however, these episodic memories decayed such that performance of controls resembled that of Jon: because neither group had access to episodic details of the learning sessions to support their semantic recall.

Another open question is whether or not the ultimate consolidated semantic memory in DA is of the same quality as that which is acquired with full support of the hippocampal system during learning. There are few data points that can inform this question. It is likely, however, that hippocampal damage has an impact on the way in which the world is explored [38], and so aspects of perceptual experience may be encoded differently in patients with hippocampal damage compared to healthy controls. In addition, the hippocampus is considered to be critical for binding unique memory items with the context in which they are encountered, while semantic memory may rely in part on linking together related semantic concepts such as objects and their associated environment. [39] studied a patient with DA using a semantic task wherein a concept (such as “squid”) was presented and the patient was asked to write down as many associated features of that concept as possible. Results showed that compared to ten controls (five of whom were university-educated), the patient wrote an equivalent number of features of the relevant concepts, indicating on the surface that her semantic understanding of the concept was unimpaired compared to controls. However, the patient wrote fewer “extrinsic” features (e.g. lives in the ocean, floats in water) than the control volunteers. If these findings are replicated using a control group that is appropriately-matched to the educational and general knowledge standards of the patient, then the following could be surmised: Where the semantic understanding of a concept depends, in part, on an association between an item and its context, then patients with early-onset hippocampal damage may develop subtly different semantic representations than controls.

## 6 Summary and conclusions

In this review, we have described the typical profile and clinical presentation of patients with DA within the context of theoretical models that account for the ontogenetic development of memory systems in relation to their neural substrates. We have highlighted that in contrast to adult-onset hippocampal amnesia, patients with early-onset DA naturally acquire a wealth of semantic knowledge outside the laboratory setting and achieve semantic memory and factual knowledge standards similar to their peers despite their severe episodic memory impairment. When these same patients are taught new information in the laboratory setting, however, they appear to learn very slowly, require many exposures to the relevant information to support their recall, and fail to reach the standards of their matched controls. This compromised pattern of learning suggests that normally, before information is consolidated into the context-free semantic system, contributions from the hippocampal-dependent episodic memory system may be necessary to support new learning. In the absence of sufficiently strong contributions from the hippocampus, the structure dedicated to fast processing of trial-unique stimuli, patients with DA must necessarily rely on direct consolidation of gist using the slow, cortically-mediated and context-free memory system. With the passage of time, however, novel factual knowledge becomes incorporated into the existing and generalised semantic system, thus approaching the standard of healthy controls. In this scenario, therefore, the hippocampus would not be necessary for the ultimate consolidation and retrieval of semantic memory in DA. At least some of the success of the compromised semantic learning system in such cases would be attributable to the putative compensatory mechanism of the immature brain that rescues, and possibly augments, non-hippocampal-dependent mnemonic processes, such as recognition and implicit retrieval.
